# Novel therapeutic approaches for the treatment of castration-resistant prostate cancer^☆^

**DOI:** 10.1016/j.jsbmb.2013.06.002

**Published:** 2013-11

**Authors:** Isabel Heidegger, Petra Massoner, Iris E. Eder, Andreas Pircher, Renate Pichler, Friedrich Aigner, Jasmin Bektic, Wolfgang Horninger, Helmut Klocker

**Affiliations:** a Department of Urology, Innsbruck Medical University, Anichstrasse 35, 6020 Innsbruck, Austria; b Department of Hematology and Oncology, Innsbruck Medical University, Anichstrasse 35, 6020 Innsbruck, Austria; c Department of Radiology, Innsbruck Medical University, Anichstrasse 35, 6020 Innsbruck, Austria

**Keywords:** AR, androgen receptor, CRPC, castration-resistant prostate cancer, ET, endothelin, IGF, insulin-like growth factor, OS, overall survival, PCa, prostate cancer, PDGFR, platelet-derived growth factor receptor, PFS, progression free survival, PSA, prostate-specific antigen, RANK-L, RANK ligand, SD, stable disease, TKI, tyrosine kinase inhibitor, VEGF, vascular endothelial growth factor, VEGFR, vascular endothelial growth factor receptor, Castration-resistant prostate cancer, Androgen receptor, Bone metastasis angiogenesis, Immunotherapy, Radiotherapy, Chemotherapy, Growth factor receptor inhibitors

## Abstract

Prostate cancer is a leading cause of cancer death in men in developed countries. Once the tumor has achieved a castration-refractory metastatic stage, treatment options are limited with the average survival of patients ranging from two to three years only. Recently, new drugs for treatment of castration-resistant prostate cancer (CRPC) have been approved, and others are in an advanced stage of clinical testing. In this review we provide an overview of the new therapeutic agents that arrived in the clinical praxis or are tested in clinical studies and their mode of action including hormone synthesis inhibitors, new androgen receptor blockers, bone targeting and antiangiogenic agents, endothelin receptor antagonists, growth factor inhibitors, novel radiotherapeutics and taxanes, and immunotherapeutic approaches. Results and limitations from clinical studies as well as future needs for improvement of CRPC treatments are critically discussed.

## Highlights

• New drugs approved for treatment of castration resistant prostate cancer.
• Prime targets: androgen receptor, bone cells, cell division, immune system.
• Several promising drugs disappointed in clinical trials.
• Further efforts necessary to optimize the sequence and combinations of drugs.
• New biomarkers required for stratification of patient and therapy selection.

## 1 Introduction

Prostate cancer (PCa) is the most frequently diagnosed malignancy in men in Western countries [1]. While localized PCa can potentially be cured by surgery or radiation therapy, metastatic PCa still remains incurable. For locally advanced or widespread disease, suppressing the tumor growth by hormone ablation therapy represents the common therapeutic option [2]. Although initial therapy mostly results in significant long-term remission, development of hormone ablation resistance is inevitable, a status named castration-resistant PCa (CRPC). In most cases, it takes about 12 to 24 months to therapy resistance [3]. At this stage of disease treatment options are very limited. Until recently, the chemotherapeutic agent docetaxel represented the treatment of choice after castration resistance emerged, prolonging the mean life span of patients for 2.9 months [4].

## 2 New Drugs for castration resistant prostate cancer

**Fig. 1:**
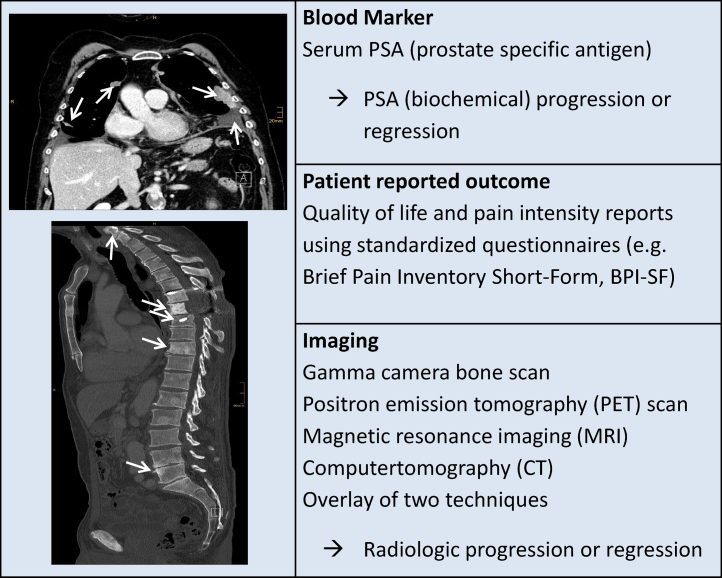
Monitoring of prostate cancer, therapy efficacy and tumor progression. Several methods are used for assessment of PCa spread, monitoring of therapy responses and determining of disease progression (right panel). The Computer tomography images (left panel) show the metastatic sites (white arrows) of patients with advanced prostate cancer.

The prostate is an androgen-dependent organ; androgen hormones and their executor, the androgen receptor (AR), are central drivers of PCa development and progression [5–10]. In hormone-naïve patients, withdrawal of androgen by surgical or chemical castration or by antiandrogens blocks AR stimulation and results in massive induction of apoptosis and tumor shrinkage. The vast majority of tumors initially respond to hormone ablative treatment, however, almost all tumors also develop resistance to this kind of therapy, after two to three years leading to further progression of the disease (disease-monitoring methods are summarized in Fig. 1) [11–13].

**Fig. 2:**
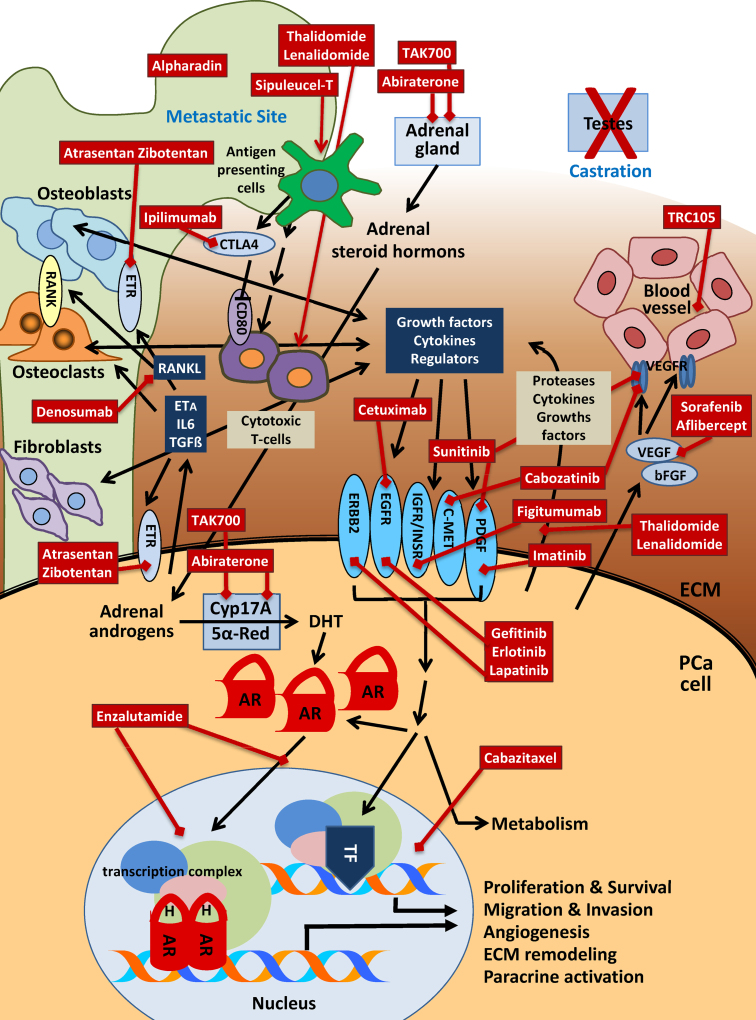
Schematic overview on new therapeutic agents for castration resistant prostate cancer (CRPC) and their targets. In metastatic CRPC testicular androgen supply is blocked by androgen deprivation therapy through chemical or surgical castration. Tumor cells (PCa) rely on the supply of weak androgen hormones from the adrenal gland, which are converted to testosterone and dihydrothestosterone (DHT) through P450 cytochrome 17,20 lyase (CYP17A) and 5α-reductase (5αRed). The androgen receptor (AR), which is often overexpressed and or mutated is activated by hormones, gain of function mutations and crosstalk with growth receptor signaling pathways and transported to the nucleus where it binds to genomic AR binding sites and initiates formation of a transcription complex and regulates genes expression. Bone is the preferred site of metastasis of prostate cancer. Prostate cancer cells release cytokines, protease and regulators to manipulate the cells in their environment (fibroblasts, osteoclasts, osteoblasts), induce angiogenesis and degrade extracellular matrix compounds (ECM) and release growth factors and compounds supporting tumor cell growth, survival and metabolism. Growth factors activate their receptors on the surface of the tumor cells to trigger intracellular signaling cascades that enhance metabolism, cell cycle progression and survival signals either directly or through stimulation of transcription factors (TF) in the nucleus. Additional players at the metastatic sites are infiltrating lymphocytes and other cells of the immune system, especially cytotoxic T-cells, which attack tumor cells. Symbols: —>, stimulation or release; —|; inhibition; , therapeutic stimulation; , therapeutic inhibition.

The combined research efforts of the last two decades boosted the insight into the mechanism of therapy resistance in PCa and provided the basis for the development of new agents (see Table 1 and Fig. 2 for an overview). The most important finding was that in the castration-resistant tumor the AR remains the key regulator and driver of tumor growth, spread and survival and the most promising therapeutic target [11]. During progression to CRPC, it adapts to the conditions of hormone ablation therapy by several mechanisms like gain-of-function mutations, expression of constitutively active receptor splice variants, receptor overexpression, alternative activation through signaling cross-talk, a change in the balance of coactivators and corepressors, recruitment of adrenal gland hormones or intratumoral de-novo androgen synthesis as alternative androgen hormone sources or downregulation of androgen metabolizing enzymes [7,12,14–17]. The advancement in understanding these molecular mechanisms of therapy resistance led to the screening for new drugs to inhibit AR signaling in the advanced cancer disease stage [18].

### 2.1 New drugs targeting the androgen receptor

One important mechanism for prostate tumors to overcome the cut-off from testicular androgen supply is intratumoral androgen production from adrenal gland androgen precursors or de-novo synthesis [17]. This mechanism is addressed with androgen synthesis inhibitors. Abiraterone acetate (Zytiga^®^) is a selective oral inhibitor of Cytochrome P450 (CYP) 17, a key enzyme in the production of androgens, estrogens and glucocorticoids within the adrenal steroid hormone synthetic pathway. CYP17 inhibition results in a further decrease of androgen levels in the circulation and in the tumors of CRPC patients [19]. CYP17 catalyzes two essential reactions in steroid hormone biosynthesis: the 17-alpha-hydroxylase reaction catalyzes the production of hormone precursors 17OH-pregnenolone and 17OH-progesterone, the C17,20-lyase activity of CYP17 then leads to the production of sex steroid precursors DHEA and androstenedione. Abiraterone acetate is a potent and selective irreversible inhibitor of both enzymatic activities [11]. After uptake, it is converted to the active compound abiraterone. In the circulation the drug is highly protein bound (>99%). It is metabolized to inactive metabolites by the cytochrome P450 oxidase CYP3A4 and the hydroxysteroid sulfotransferase SULT2A1, mainly in the liver. Tracer studies identified abiraterone sulfate and N-oxid aberaterone sulfate as the main inactive metabolites [20,21].

In preclinical studies, Abiraterone acetate has been shown to inhibit the ACTH-mediated stimulation of adrenal cells followed by a significant dose-dependent decrease in circulating testosterone levels [22,23]. Therefore continuous CYP17 inhibition induces secondary hormone ablation responses in CRPC patients. Several studies evaluated Abiraterone acetate in chemotherapy-naïve and in docetaxel-pretreated CRPC patients with response rates from 45 to 75%. The encouraging data from these studies were followed by a multicenter study evaluating the therapeutic effect of the drug in combination with prednisone given as a replacement for cortisol, whose synthesis is also blocked by Abiraterone acetate. This study reported an overall survival (OS) of 14.8 months in treated patients compared to an OS of 10.9 months in patients receiving prednisone alone. Moreover, Abiraterone acetate showed improvements of progression free survival (PFS) of 5.6 vs. 3.6 months and PSA response rates of 29.1 vs. 5.5% compared to prednisone [24]. The drug was generally well tolerated with only mild side effects.

In 2011, Abiraterone acetate (Zytiga^®^) in combination with prednisone has finally been approved for the treatment of patients with CRPC who have received prior docetaxel chemotherapy. A subsequent study focused on treatment of chemo-naïve patients (COU-AA-302). In these patients, the median radiographic PFS was 16.5 months with Abiraterone acetate-prednisone and 8.3 months with prednisone alone. Furthermore, with Abiraterone acetate-Prednisone OS improved and delayed subsequent chemotherapy and cancer-related pain [25]. Based on these, data Abiraterone acetate has been approved for treatment in the pre-chemotherapy phase of CRPC in 2013.

Besides surgical, LHRH agonist and antagonist inhibition of testosterone biosynthesis non-steroidal antiandrogens like flutamide (Eulexin^®^) and bicalutamide (Casodex^®^) which inhibit the AR transactivation function belong to the standard therapeutic armamentarium for treatment of advanced PCa and are also used in combination with anti-LHRH treatment to achieve complete androgen blockade and prevent the initial flare-up phenomenon of LHRH agonists [4,26]. These antiandrogens have a low affinity for the AR and can lose their AR inhibitory property or even become AR agonists in castration-resistant tumor cells [27,28]. A reason for that may be the corruption of the AR inhibitory effect by interaction of AR with pioneering transcription factors like FoxA1. FOXA1 binding reorganizes chromatin and makes it more accessible for AR binding, thus, modulating the AR response [29]. This transcription factor was shown to shift the antagonist/agonist balance of bicalutamide toward an agonist response [30]. New next-generation AR antagonists have been developed for use in CRPC patients that have a higher affinity for the AR with less agonistic potential and enhanced inhibitory efficacy.

The first representative of next-generation antiandrogens to enter the clinics is Enzalutamide (Xtandi^®^, MDV3100), a selective AR antagonist that inhibits AR signaling by preventing nuclear translocation of the ligand-receptor complex and its transformation to a transactivation-competent transcription factor, thereby blocking the AR from regulating its target genes [31]. Results from early clinical studies showed a substantial antitumor activity and significant PSA decrease under MDV3100 therapy in CRPC patients with and without previous chemotherapy [32,33]. Two large phase III clinical studies, AFFIRM and PREVAIL (the latter is still ongoing), were initiated to test MDV3100 efficacy in patients who received prior docetaxel therapy and in chemotherapy-naïve patients, respectively. The final outcome of the AFFIRM trial has been published recently showing that MDV3100 prolonged OS, slowed disease progression, and improved quality of life in CRPC post-docetaxel treatment [34]. The median OS was 18.4 months with MDV3100 compared to 13.6 months in the placebo group, and the radiographic PFS improved from 2.9 to 8.3 months. This finding led to approval of MDV3100 for post chemotherapy treatment by the FDA.

Orteronel (TAK-700) was originally known as VN/124-1 and is an oral, selective, reversible, non-steroidal androgen synthesis inhibitor. It inhibits only one of the two enzymatic reactions catalyzed by CYP17, the 17,20 lyase activity. Due to its low inhibition of 17-hydroxylase activity, it has a negligible effect on glucocorticoid synthesis and its administration does not require concomitant cortisol replacement [35]. A recent study quantified the inhibitory activity and specificity of TAK-700 for testicular and adrenal androgen production by evaluating its effects on CYP17A1 enzymatic activity and showed potent inhibition of 17,20-lyase activity in monkey adrenal cells and human adrenal tumor cells and reduction of circulating testosterone levels in monkeys [36]. After successful phase I and phase II studies to date, two phase III studies evaluating the effect of TAK-700 in combination with prednisone are ongoing [37,38].

While the new AR targeting drugs are introduced into clinical practice, preclinical studies continue to better understand their molecular function and the development and possible prevention of resistance mechanisms. Despite therapeutic efficacy in the majority of CRPC patients, it also became clear that primary and acquired resistance to this drug occurs, mostly accompanied by increasing PSA levels suggesting resumed AR signaling. Several possible reasons for this, like up-regulation of CYP17A1, constitutive gain-of-function mutations of the AR through generation of truncated splice variants or activation of a mutated promiscuous AR by endogenous or exogenous steroids have been shown but remain to be elucidated in detail [39]. Moreover, there is as good rationale to evaluate a combined treatment with substances providing complementary AR inhibition such as Abiraterone acetate and Enzalutamide [11,39] or to combine the new drugs with recently defined inhibitors of constitutively active variant ARs [40].

### 2.2 Agents targeting bone metastases and environment

Bone metastases represent the most common type of metastases in PCa. Therefore the bone represents an important therapeutic target in advanced stages of PCa. It is a dynamic tissue that undergoes constant remodeling by osteoclasts and osteoblasts. This environment is the ideal “soil” for tumors such as breast and prostate [41]. Tumor cells disturb the balance of osteoblast and osteoclast activity by secretion of bone-active regulatory factors such as endothelin-1 (ET1), interleukin-6 (IL6) and transforming growth factor β (TGFβ) in order to enhance bone turnover and release of nutrients and growth factors. A crucial regulator in bone physiology is the RANK ligand (RANK-L). Under physiological conditions it is mainly produced by osteoblasts and binds to its receptor RANK on the surface of monocytes and osteoclasts, thereby, stimulating osteoclast formation, differentiation and activation. RANK-L is increased in metastatic lesions of prostate cancer resulting in bone destruction or pathological bone formation [42,43].

Denosumab (Xgeva^®^) is a fully humanized monoclonal antibody that specifically inhibits osteoclast activity by binding to RANK-L, thereby, modulating intracellular signaling pathways involved in osteoclast formation, their function and survival. In a large phase III multicenter study (HALT) comparing Denosumab with bisphosphonate zoledronic acid, the standard treatment option for deferring development and progression of bone metastases in CRPC, the median time to bone metastasis was 20.7 months versus 17.1 months, respectively [44]. In 2010, Denosumab was approved for treatment of skeletal-related events in solid tumors including PCa.

Takeup of salts for bone mineralization is utilized to guide new emitters to the bone. Radium-223 chloride (Alpharadin^®^) is an injectable form of an alpha particle emitting radium-223 salt seeking bone [45]. First clinical studies reported a reduction in bone metastasis markers and PSA reduction as well as improved OS after Alpharadin therapy [45]. Currently a phase III double-blind, randomized, multinational study (ALSYMPCA) is comparing treatment with Alpharadin vs. placebo in CPRC patients with bone metastases. A recently presented analysis of the study showed improved OS with a highly favorable safety profile. The median OS was 14 months in the Alpharadin group compared to 11.2 months in the placebo group [46].

Endothelins (ETs) are vasoconstricting 21-amino acid peptides that also stimulate the release of factors involved in the regulation of bone homeostatis. In particular, ET-1 is released by prostate tumor cells and plays a crucial role in PCa development and progression through induction of cell proliferation, survival, angiogenesis and finally formation of bone metastases [47,48]. The function of endothelins is mediated through two receptors, ET-A and ET-B. Preclinical studies found that ET receptor levels on tumor cells correlate with tumor stage, tumor grade and metastases. Moreover, endothelin receptor A is also highly expressed in bone osteoblasts and osteoclasts and endothelin stimulates new bone formation and development of osteoblastic bone metastases. Consequently ET receptors seem promising anticancer targets [47,49,50].

Atrasentan is a selective ET-A antagonist; it blocks ET-A with a 1800-fold higher selectivity than ET-B. In a phase II study performed in CRPC patients, Atrasentan prolonged the time to disease progression and PSA progression significantly in comparison to the placebo [51]. However, in a subsequent phase III study involving 809 patients, Atrasentan failed to reduce the risk of disease progression. Nevertheless, quality of life and pain scores as well as alkaline phosphatase (AP) and PSA levels improved [52]. Recently, the SWOG S0421 phase III study comparing docetaxel plus prednisone vs. the addition of Atrasentan to these drugs was stopped by the Data and Safety Monitoring Committee because neither OS nor PFS were significantly improved [38].

Zibotentan (ZD4054) is another highly selective ET-A antagonist. Although it could not show improvement of disease progression in comparison to the placebo, OS was significantly longer in the Zibotentan arm in an initial safety and efficacy study [53,54]. In phase III studies, Zibotentan did not show a significant benefit in CRPC patients or in patients with biochemical progression [55,56]. Still under way are studies comparing Doxetacel treatment with or without addition of Zibotentan [38]. A preliminary analysis of a small tolerability and efficacy study showed some benefit for this combination [57].

### 2.3 Immunotherapy and immunomodulators

The concept of training the immune system to overcome tumor-induced tolerance using vaccinations against tumor antigens is successfully used in the treatment of several cancer entities. Immunotherapeutic approaches aim to modulate immunostimulatory pathways that help to maintain and to prolong the activity of antigen-presenting cells and enhance cytotoxic T-cell-mediated tumor regression [58,59]. Recent evidence suggests that PCa is more immunogenic than previously thought [59,60]. Thus, a number of immunological anticancer strategies are currently under investigation.

Sipuleucel-T (Provenge^®^) is an autologous vaccine consisting of patients’ autologous peripheral blood mononuclear cells stimulated ex vivo to generate antigen-presenting cells. The recombinant stimulatory protein used consists of the target antigen prostatic acid phosphatase (PAP) fused to granulocyte macrophage colony stimulating factor (GM-CSF). After reinfusion of the stimulated autologous antigen presenting cells into the patient an immune response to PAP-expressing PCa cells is induced. Sipuleucel-T is administered to patients as a freshly manufactured preparation by intravenous injection. Standard treatment consists of three infusions [61]. Although in a phase III study, the median time to progression of PCa patients under Sipuleucel-T therapy did not differ from placebo-treated patients, the OS was in favor of those patients with immunotherapy (25.8 months vs. 21.7 months) [61,62]. In 2010, Sipuleucel-T was approved by the FDA for the treatment of asymptomatic or minimally symptomatic CRPC. The limited availability especially outside the US and the high therapy costs currently restrict Sipuleucel-T as a standard treatment option for CRPC.

Prostvac-VF is a recombinant vaccinia-based viral construct encoding PSA and three immune co-stimulatory molecules (intracellular adhesion molecule-1, B7-1 and leukocyte function-associated antigen-3) [63]. In a randomized phase II study involving 125 patients with minimally symptomatic CRPC, the median PFS (the primary endpoint) was similar in the group receiving Prostvac-VF to that in the group receiving control vectors. At three years post-study, the median survival in the Prostvac-VF group was significantly longer (21.5 vs. 16.6 months for controls) with an estimated HR of 0.56 (95% CI 0.37, 0.85; *p* = 0.061) [62]. A phase III study is currently ongoing [38].

Another immune-modulating approach is the blockade of cytotoxic T-lymphocyte-assiociated antigen 4 (CTLA-4) using the monoclonal humanized antibody Ipilimumab (MDX-101, Yervoy^®^), which was approved in 2011 for the treatment of late stage-melanoma [64]. The antibody target CTLA-4 is a co-stimulatory molecule that binds to CD80 (B7) on the surface of T-lymphocytes and functions as a negative regulator of T-cell activation as part of the system regulating an immune response. Trapping of CTLA4 by Ipilimumab enhances the cytotoxic T cell response against tumor cells [65]. Recently a phase III study in chemo-naive PCa (CA184-095) and another phase III study in docetaxel-pretreated patients have been started (CA184-043) [38]. In addition, approaches to combine Ipulimumab stimulation of an immune response with vaccination is a promising approach tested for the treatment of CRPC and other tumors [65].

Thalidomide and its second-generation analog Lenalidomide are immunomodulatory and antiangiogenic compounds targeting both cancer cells and their microenvironment. While their antiangiogenic activity is due to inhibition of the secretion of VEGF, bFGF, Interleukin-8 or TNFα and fibroblast growth factor (FGF) from tumor and tumor stroma cells, their immunomodulatory effect is caused by stimulation of T-cells and natural killer cells as well as the inhibition of T-regulatory cells [59].

In phase II studies, Thalidomide treatment in combination with docetaxel chemotherapy showed a decline of serum PSA values, and an improvement of OS and PFS. Treatment with combinations of Docetaxel plus Thalidomide plus Bevacizumab (an antiangiogenetic monoclonal antibody, see the next chapter) even showed PSA responses in 80% of the patients [66]. However, Thalidomide treatment was accompanied by toxic side effects including deep venous thrombosis, constipation or neuropathies.

To reduce the side effects of these therapies, a second-generation analog named Lenalidomide with a more favorable neurotoxic profile has been developed. In a phase I/II study, Lenalidomide monotherapy showed stable disease in 63% and PSA responses in 38% of CRPC patients. Combination therapy of Lenalidomide plus Docetaxel caused PSA declines of >50% in half of the treated patients [67]. To date, several studies investigating Lenalidomide in combination with chemotherapy, with GM-CFS or with Cyclophosphamide are ongoing [38]. The combined therapy with Lenalidomide and Bevacizumab plus Doxetacel and prednisone was associated with high PSA (85.2%) and tumor (86.7%) responses in metastatic CRPC, with manageable toxicities [65,68].

### 2.4 Inhibitors of angiogenesis

Tumor growth beyond a size of 3 mm is dependent on the development of new blood vessels, a process called angiogenesis [69]. One of the key players during angiogenesis is the vascular endothelial growth factor A (VEGF-A), which signals through VEGF receptors (VEGFR) [70]. Tumor cells enhance local VEGF production to stimulate the outgrowth of new blood vessels; moreover elevated VEGFR levels are associated with cancer progression and poor survival rates. Thus, targeting the VEGF pathway represents an attractive anti-cancer approach. Several approaches are employed to inhibit the VEGF pathway either by targeting VEGF itself, or the VEGF receptors or by targeting downstream signals in the pathway [70].

Bevacizumab (Avastin^®^) is a monoclonal antibody that potently inhibits VEGFR signaling through binding and neutralizing VEGF-A. Currently Bevacizumab is approved for the treatment of several cancer entities including colorectal cancer or breast cancer. Although phase II studies in PCa, where Bevacizumab has been administered in combination with chemotherapy, showed encouraging results, Bevacizumab has not been successful in a phase III setting [71]. The combination with docetaxel and prednisone did not result in any OS benefit in comparison to docetaxel and prednisone alone. Moreover, addition of Bevacizumab was associated with higher treament-related toxicity and with an increased number of treatment-related deaths [72].

Sorafenib (Nexavar^®^) is a multi-tyrosine kinase inhibitor that decreases tumor growth and disrupts tumor microvasculature through inhibition of multiple targets including the VGEF receptors VEGFR-1, VEGFR-2 and VEGFR-3 as well as Raf serine/threonine kinases and platelet-derived growth factor receptor β (PDGFR) [73]. Thus, besides angiogenesis Sorafenib also targets growth factor pathways. Currently, Sorafenib is clinically approved for the treatment of several cancer entities like renal or hepatocellular cancer [73]. Phase II studies in CRPC observed therapeutic activities of Sorafenib, such as prevention of radiologic progression and regression of bone metastases. However, no PSA decline was observed under Sorafenib therapy. Therefore the investigators of these studies concluded that PSA measurement might not be an accurate marker of therapy response under Sorafenib treatment [74]. Another phase II study of sorafenib in combination with bicalutamide in patients with chemotherapy-naive CRPC reported a PSA response or stable disease for 6 months or longer in 47% of the patients. Serum PSA declines of ≥50% occurred in 32% of patients. The median time to treatment failure was 5.5 months [75]. Currently, Sorafenib monotherapy is evaluated in a phase III study of patients in docetaxel-refractory PCa patients [38].

Aflibercept (Eylea^®^) is an anti-VEGF agent representing a VEGF-trap. It is a recombinant protein consisting of the Fc portion of human IgG1 combined with the extracellular ligand-binding domains 2 and 3 of the human VEGFR 1 and 2 which functions as a decoy receptor for VEGFs [76]. Aflibercept is currently under investigation in phase III (VENICE trial) in combination with first-line docetaxel, treatment [38].

Another multi-tyrosine kinase inhibitor is Sunitinib (Sutent^®^) which inhibits the tyrosine kinase receptors VEGFR, platelet derived growth factor receptors (PDGFR) and c-kit. Sunitinib is approved for treatment of renal cell cancer, gastrointestinal stromal tumors or pancreatic neuroendorine tumors [77]. Zurita et al. analyzed Sunitinib plus prednisone and doxetacel in CRPC patients in a phase I/II study and found that the combination of all three agents is well tolerated and has substantial benefits regarding response rates and OS benefits [78]. However, a phase III study investigating sunitinib plus prednisone in patients with metastatic CRPC after failure of docetaxel chemotherapy (SUN 1120) with OS as the primary endpoint was prematurely discontinued recently due to lack of efficacy [38].

A protein that is essential for angiogenesis and vessel development and, thus, a promising anti-vascular target is Endoglin/CD105 [79]. It is a major glycoprotein of the vascular endothelium forming a homodimeric transmembrane complex that binds TGFβ-1 and -3 with high affinity. It participates in transforming growth factor beta receptor signaling. An anti-endoglin monoclonal antibody (TRC105) was tested in a variety of solid tumors. Ongoing clinical trials are testing it in combination with chemotherapy or VEGF inhibitors or as a single agent in prostate, ovarian, bladder, breast, and hepatocellular cancer [80].

### 2.5 Growth factor receptor inhibitors

Growth factors stimulate proliferation, support survival and enhance migration and invasion of prostate cancer cells [49]. Growth factors bind to and activate protein tyrosine kinase receptors on the cell surface, which trigger the intracellular signaling systems [49]. These signaling cascades are major drivers of carcinogenesis, tumor progression, metastatic spread and development of resistance to tumor therapies and are of particular interest as therapeutic targets. In PCa, epidermal-, fibroblast-, PDGF-, and IGF-systems were reported deregulated either at the growth factor or the receptor levels or both. New molecular therapeutics have been developed for inhibition of the peptide growth factors themselves, or for blocking their receptors or intracellular signaling components or inhibiting the tyrosine kinase activity of growth factor receptors. They have been tested in different tumor identities either alone or in different combination therapies. With regard to prostate cancer, the therapeutic effects achieved in clinical trials have been modest so far.

Epidermal growth factor receptor (EGFR) targeting agents are successfully used in different cancer entities like lung or breast cancer. In PCa, Gefitinib (Iressa^®^) an EGFR tyrosine kinase inhibitor (TKI), however, failed to demonstrate PSA declines or clinical responses when given as a monotherapy in CRPC patients [81,82]. Likewise, combination therapies of Gefininib and Docetaxel did not improve OS or PFS. The EGFR TKI Erlotinib (Tarceva^®^) exerted a moderate activity in chemotherapy-naive, CRPC patients, with some patients showing a PSA response [38]. Currently the EGFR and ERBB2 (HER-2) dual TKI Lapatinib (Tyverb^®^) is under clinical investigation. Phase II studies so far indicate that it may have some efficacy in men with CRPC although only few PSA responses were observed [83,84].

The chimeric monoclonal antibody Cetuximab (IMC-A12, Erbitux^®^) binds to EGFR and prevents its intracellular signaling. Currently, it is approved for treatment of wild-type KRAS colon and head and neck cancer. The combination of cetuximab with mitoxantrone plus prednisone was evaluated in post-docetaxel therapy CRPC patients in a phase II study including 115 patients. The observed effects did not support the use of cetuximab in this drug combination, but it might still be of use in other settings [85].

The TKI Imatinib (Gleevec^®^) was developed for targeting the fusion kinase Bcl-Abl, which plays a central role in leukemia. It also inhibits the kinase activities of other receptors like c-Kit, the receptor for hepatocyte growth factor, or PDGF receptor [49]. Imatinib monotherapy studies and small combination studies with docetaxel or the antiangiogenic drug Sorafenib in PCa patients are disappointing so far [86].

Inhibitors of the insulin-like growth factor receptor-1 (IGF1R) are studied in a number of different tumor types including PCa [87–89]. Administration of the anti-IGFR1 monoclonal antibody Figitumumab (CP-751,871) a fully humanized IgG2 monoclonal antibody in combination with docetaxel showed radiographic responses in 22% and SD for more than 6 months in 11% of CRPC patients [90]. Further clinical studies are under way [38].

Cabozantinib (XL184, Cometriq™) is an oral small molecule inhibitor of multiple kinase signaling pathways including c-MET and VEGFR2. In phase I clinical studies, Cabozantinib resulted in tumor regression in multiple cancer types [91]. A recently published phase II study compared response rates at 12 weeks and PFS after random assignment of either Cabozantinib or placebo. Median PFS was 23.9 weeks in the treatment and 5.9 weeks in the placebo group [92]. Cabozantinib treatment resulted in stable disease in 75% and objectives response rates in 5% of treated patients. A phase III, randomized, double-blind, controlled study of Cabozantinib versus prednisone in metastatic CRPC patients who have received prior docetaxel and Abiraterone acetate or MDV3100 is under way. A second phase III study is comparing Cabozantinib versus Mitoxantrone plus prednisone in men with previously treated symptomatic CRPC [38].

### 2.6 New taxanes

Cabazitaxel (Jevtana^®^) is a novel taxane that showed activity in docetaxel-resistant tumor cell lines [93]. Recently, cabazitaxel was reported to have an OS benefit in patients with CRPC who have progressed in docetaxel therapy when compared to mitoxantrone chemotherapy (TROPIC trial) [93]. Median progression-free survival was 2.8 months in the cabazitaxel group and 1.4 months in the mitoxantrone group. Like other taxanes, cabazitaxel exerts its cytotoxic effect through mitotic arrest at the metaphase-anaphase transition, ultimately leading to cell death. Unlike docetaxel that has an affinity for multidrug resistance (MDR) proteins, which is as a major mechanism of resistance, cabazitaxel demonstrated poor recognition by MDR proteins [93]. On the basis of this finding, cabazitaxel is indicated for the treatment of patients with metastatic CRPC who have previously been treated with docetaxel. In 2010, Cabazitaxel was approved by the FDA. Even through toxicity is still a matter of concern, a recent safety report based on 111 treated patients concluded that side effects are tolerable [94].

## 3 Conclusion

In the last few years, many different therapeutic strategies for CRPC have been developed and evaluated in clinical studies. Several strategies showed objective clinical benefit and have been approved for clinical use. On the other hand, there are several examples of new molecular targeting drugs that showed promising results in preclinical PCa models but showed insufficient effects in clinical phase III studies. This raises the question of how preclinical models and preclinical testing can be improved to avoid such failures in the future.

The expanded arsenal of drugs now available for treatment of CRPC denotes a big step forward to tailor treatment for each patient and to prolong control of the disease. The newly approved drugs can retard disease progression by several months but still cannot prevent it. Further efforts are necessary to optimize the sequence of use and the best combinations of the different drugs in order to optimize and further enhance PFS and OS survival and quality of life of affected patients. Many of the newly available molecular therapeutics in the pipeline still have to prove their efficacy in clinical praxis. Another challenge is establishment of selection criteria to define those patients who are likely to respond to a certain type of therapy. This means there is also a need for appropriate biomarkers and companion diagnostics to define patient populations that will benefit from specific treatments.

## Figures and Tables

**Table 1:** Summary of new drugs for castration resistant prostate cancer.

Agent	Target	Approved for cancer treatment	Study phase prostate cancer	References clinical studies
Drugs targeting the androgen receptor activity				
Abiraterone acetate	CYP17 enzyme	Yes	Approved 2011	[18,19]
Enzalutamide	Androgen receptor	Yes	Approved 2012	[24]
Oteronel	CYP17 enzyme	No	III	[35,36]

Drugs targeting bone metastasis and environment				
Denosumab	RANK-L	Yes	Approved 2010	[42]
Alpharadin	Tumor cells in bone	No	III	[43,44]
Atrasentan	Endothelin receptor A	No	II	[49]
Zibitentan	Endothelin receptor A	No	III	[53–55]

Immunotherapy and immunomodulators				
Sipuleucel-T	APC-antiPAP	Yes	Approved 2010	[59,60]
Prostavac	APC-antiPSA	No	III	[61]
Ipilumumab	CTLA-4	Yes	III	[63]
Thalidomide	NK cells, regulatory T-cells VEGF, bFGF, IL-6, TNFα	Yes	II	[57]
Lenalidomide	VEGF, bFGF, Interleukin-8, TNFα	Yes	I/II	[57]

Inhibitors of angiogenesis				
Bevacizumab	VEGF	Yes	III	[70]
Sorafenib	VEGFR	Yes	III	[73]
Sunitinib	VEGFR	Yes	III	[37]
TRC105	Vascular endothelium	No	I/II	[77]
Aflibercept	VEGF	Yes	III	[74]

Growth factor receptor inhibitors				
Gefitinib	EGFR	Yes	II	[79,80]
Erloninib	EGFR	Yes	II/III	[38]
Lapatinib	EGFR, ERBB2	Yes	II	[81,82]
Imatinib	PDGFR	Yes	II	[84]
Figitumumab	IGF1R	No	II	[88,37]
Cetuximab	EGFR	Yes	II	[90]
Cabozantinib	cMET, VEGFR2	Yes	III	[90]

New Taxanes				
Carbazitaxel	Tumor cell division	Yes	Approved 2010	[91]

Overview on new therapeutic agents for CRPC that have been recently approved or are in clinical trials and their mode of action and direct targets.
